# Methamphetamine-withdrawal catatonia resolved with mixed amphetamine salts: a case report

**DOI:** 10.3389/fpsyt.2026.1947002

**Published:** 2026-09-22

**Authors:** Elischa J. Sanders, Stephanie C. Valcourt

**Affiliations:** 1 Massachusetts General Hospital, Boston, MA, United States; 2 Psychotic Disorders Division, McLean Hospital, Belmont, MA, United States; 3 Department of Psychiatry, Harvard Medical School, Boston, MA, United States

**Keywords:** Bush-Francis Catatonia Rating Scale, case-report, methamphetamine-withdrawal catatonia, mixed amphetamine salts, treatment-resistant catatonia

## Abstract

Catatonia is a well-recognized psychiatric emergency with an evolving understanding of its underlying pathophysiology. However, withdrawal-associated catatonia remains poorly characterized. Here we describe a 32-year-old man with symptoms consistent with methamphetamine-withdrawal catatonia, a previously undescribed etiology, which resolved only after treatment with mixed amphetamine salts. The patient remained catatonic (Bush-Francis Catatonia Rating Scale [BFCRS] >10) for four months despite 38 sessions of electroconvulsive therapy, lorazepam up to 24 mg daily, and multiple augmentation strategies. An extensive medical and neurologic workup excluded alternative causes. Following initiation of mixed amphetamine salts, his BFCRS decreased from 15 to 6 within 48 hours, with sustained resolution and no recurrence of psychosis despite discontinuation of antipsychotics. This case suggests that methamphetamine withdrawal may represent an underrecognized cause of catatonia and that dopaminergic augmentation may have a therapeutic role in refractory cases.

## Introduction

Catatonia is a neuropsychiatric syndrome and a recognized psychiatric emergency associated with significant morbidity and mortality when left untreated (1–3). Historically characterized by motor, behavioral, and affective disturbances, it is increasingly understood as a disorder involving dysregulation across multiple neural circuits, including GABAergic, dopaminergic, and glutamatergic pathways (4). Although its precise pathophysiology remains incompletely elucidated, first-line treatment with benzodiazepines—most commonly lorazepam—and electroconvulsive therapy (ECT) in refractory cases is highly effective for most patients (4, 5).

Despite these established treatments, a subset of patients exhibit persistent or refractory catatonia, posing substantial clinical challenges. Here, we present a case of severe, treatment-resistant catatonia in which high total daily doses of lorazepam and more than 30 sessions of ECT failed to produce sustained clinical improvement. Notably, resolution of catatonic symptoms was achieved only after the addition of mixed amphetamine salts (immediate- and extended-release formulations), a therapeutic approach not previously described in the treatment of catatonia.

While stimulant-associated (particularly methamphetamine-induced) catatonia has been reported in the literature (6, 7), this case suggests a novel mechanism—catatonia emerging in the context of methamphetamine withdrawal. The patient’s clinical course and response to dopaminergic augmentation raise the possibility of withdrawal-related hypodopaminergic states contributing to catatonic pathophysiology (8–10).

Given the potentially life-threatening nature of catatonia, along with the rising prevalence of methamphetamine use in the United States (11–13), this case highlights the importance of recognizing atypical etiologies and considering alternative treatment strategies in refractory presentations.

## Patient information and history

We present a case of a 32-year-old college-educated white male with a psychiatric history of schizoaffective disorder (depressive type), generalized anxiety disorder, and opioid use disorder in remission, with no significant medical history, who presented with worsening psychosis in the setting of recent methamphetamine use.

The patient’s home medication regimen consisted of risperidone 150 mg long-acting injectable that patient received one time, bupropion standard release 100 mg daily, baclofen 20 mg three times per day, and buprenorphine-naloxone 8 mg twice per day.

Regarding substance use, opioid and marijuana use were in remission. However, the patient regularly consumed methamphetamine. Prior to admission, he reported smoking between 3–7 g of methamphetamine over the course of three days, which significantly exceeded his baseline of approximately 2 grams per month over the preceding six months. He had previously experienced hallucinations that made him fear for his life, leading to multiple suicide attempts and inpatient hospitalizations.

## Additional neurologic and medical workup

An extensive medical and neurologic workup was conducted to evaluate alternative causes of catatonia. Laboratory evaluation included testing for metabolic and hematologic abnormalities, syphilis, HIV, thyroid dysfunction, Wilson’s disease, lupus (C3, C4, dsDNA ab, SS-B(La), Sm Ab) and autoimmune encephalitis (20 markers including: AMPA-receptor antibody, Amphiphysin antibody, AGNA-1, anti-neuronal nucleus antibody, ANNA-2, ANNA-3, CASPR2-IgG cell based assay, CRMP-5-IgG, GABA B receptor antibody, GAD65 antibody, GFAP immunoflourescnce assay, LGI1-IgG cell based assay, mGluR1 antibody, NMDA antibody). As recommended by neurology consults, two brain MRIs were obtained, which were unremarkable.

## Treatment and hospital course

On admission, the patient reported using methamphetamine four days prior. The patient described using methamphetamine to enter grandiose, fantasy-like states in which he felt more capable of pursing idealized versions of his goals, which included improving both interpersonal and romantic relationships. His initial presentation was notable for subacute worsening of psychotic symptoms, including self-dialoguing, auditory hallucinations, thought permeability, experiences of external control, and delusional preoccupation with religious punishment. (**Table 1**).

**Table 1:** Timeline of clinical course showing events prior to admission through discharge.

Timepoint	Clinical status/BFCRS score	Intervention	Outcome
Pre-admission	Increasing methamphetamine use from ~ 2 grams/month to 3-7 grams in four days prior to admission	Home medications: Risperidone LAI 150 mg, bupropion SR 100 mg daily, baclofen 20 mg TID, buprenorphine-naloxone 8 mg BID	Worsening psychosis
Admission	Brought to hospital by family for erratic behavior and hallucinations	Inpatient admission start lorazepam 2 mg nightly	Catatonia emerges shortly after admission
Early course Days 0-2	BFCRS > 10, features include: waxy flexibility, gegenhalten, staring, perseveration, and echophenomena	Lorazepam increase: 2 mg nightly to 12 mg TDD	Catatonia slightly improved, waxy flexibility, gegenhalten and perseveration remain
Early course Days 5-10	BFCRS > 10, features include: waxy flexibility, gegenhalten, and perseveration	Clozapine initiated and titrated to 37.5 mg Lorazepam increased to 18 mg TDD	Catatonia improved Clozapine discontinued for concern of myocarditis (fever, tachycardia, elevated CRP)
Early course Days 12-29	BFCRS ~ 9, features include: waxy flexibility, and gegenhalten	Bilateral ECT started 3x/week Lorazepam 18 mg TDD	BFCRS ~ 6, features include: waxy flexibility
Early course Days 30-44	BFCRS ~ 6, features include: waxy flexibility	Bilateral ECT continued 3x/week Attempted to decrease lorazepam Lowest dose achieved over one week 10.5 mg TDD	Catatonia worsens BFCRS ~ 12, features include: waxy flexibility, gegenhalten, ambitendency, and posturing
Mid course Days 45-73	BFCRS > 10, features include: waxy flexibility, gegenhalten, and perseveration	Memantine 5 mg BID started Lorazepam increased to 24 mg TDD Bilateral ECT continued 3x/week	Catatonia improved → BFCRS ~6 waxy flexibility persisted Memantine discontinued because patient developed a rash
Mid course Days 74-115	Catatonia worsens BFCRS > 10, features include: waxy flexibility, gegenhalten, ambitendency, and posturing	Lorazepam 24 mg TDD Bilateral ECT continued 3x/week	Catatonia persists, refractory to treatment BFCRS > 10, features include: waxy flexibility, gegenhalten, ambitendency, and posturing
Mid course Days 116-122	Catatonia persists, refractory to treatment BFCRS > 10, features include: waxy flexibility, gegenhalten, ambitendency, and posturing	Amantadine 50 mg BID started Lorazepam 24 mg TDD Bilateral ECT continued 3x/week	Catatonia persists, refractory to treatment BFCRS > 10, features include: waxy flexibility, gegenhalten, ambitendency, and posturing
Late course Days 123	Catatonia persists, refractory to treatment BFCRS > 10, features include: waxy flexibility, gegenhalten, ambitendency, and posturing	Trial of intravenous lorazepam started Amantadine 50 mg BID continued Lorazepam 24 mg TDD Bilateral ECT 38/38 complete	Catatonia persists, refractory to treatment BFCRS > 10 Patient cannot tolerate IV
Late course Days 124-126	Catatonia persists, refractory to treatment BFCRS > 10, features include: waxy flexibility, gegenhalten, ambitendency, and posturing	Mixed amphetamine salts-ER 5 mg daily Amantadine 50 mg BID continued Lorazepam 24 mg TDD	Catatonia improved BFCRS 15 → 6
Late course Days 126-131	Catatonia improving BFCRS ~ 6, features include waxy flexibility and gegenhalten	Zolpidem 5 mg initiated Mixed amphetamine salts-ER 5 mg daily Amantadine 50 mg BID continued Decreasing lorazepam	Catatonia improving BFCRS ~ 3, features include waxy flexibility
Late course Days 131-discharge	Catatonia improving BFCRS 2 Stopped scoring catatonia	Mixed amphetamine salts-IR 10 mg initiated Zolpidem increased to 15 mg Mixed amphetamine salts-ER 5 mg daily Amantadine 50 mg BID continued Decreasing lorazepam	Catatonia resolved
Discharge	Catatonia resolved Plan for outpatient benzodiazepine and amphetamine salts taper at outpatient rehab facility	Mixed amphetamine salts-IR 10 mg Mixed amphetamine salts-ER 5 mg daily Lorazepam 4 mg TDD Zolpidem 15 mg TDD Amantadine 50 mg BID	Discharged to outpatient rehab facility Plan to decrease lorazepam 0.25 mg/week Amphetamine salts taper managed by outpatient dual-diagnosis psychiatrist

Table color key.

⬛ – Early course persistent catatonia

⬛ – Worsening catatonia, refractory to treatment

⬛ – Improving and resolved catatonia

Following admission, the patient developed severe catatonic features, including prominent waxy flexibility, gegenhalten, ambitendency, posturing/catalepsy, staring, perseveration, and intermittent verbigeration and echophenomena. He subsequently experienced four months of treatment-resistant catatonia, during which waxy flexibility, gegenhalten, and verbigeration remained persistent despite the treatment course outlined below.

Initially, the patient received 2 mg nightly of oral lorazepam though quickly required increasing doses of oral lorazepam to treat catatonia symptoms. Two days following admission the frequency increased to 2 mg every six hours. While the patient never experienced sedation with increasing frequency of medication, 12 mg of total daily dose oral lorazepam remained insufficient. The following six weeks multiple interventions and augmentations were pursued as described below (**Figure 1**).

**Figure 1:**
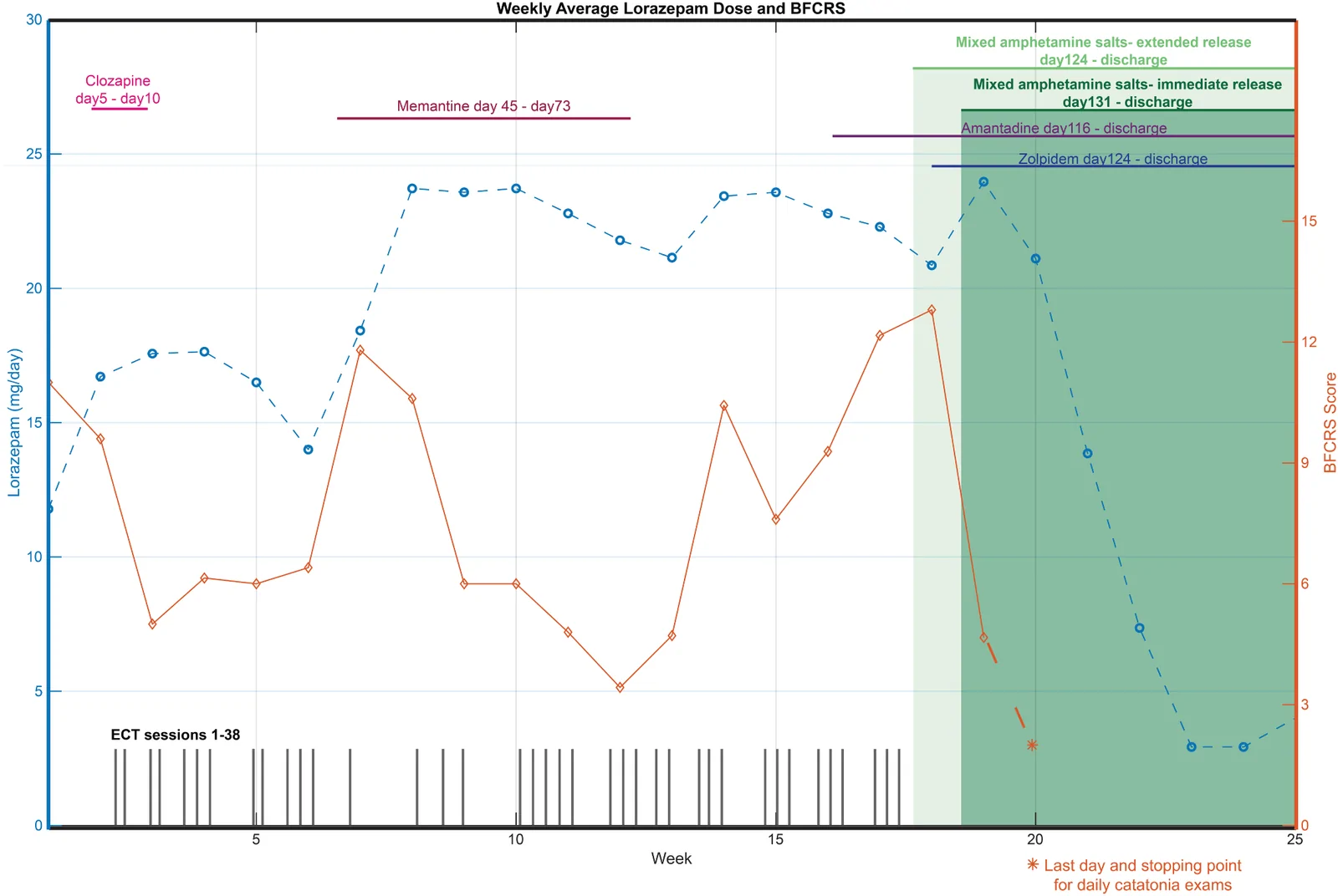
Weekly average lorazepam dose and Bush-Francis Catatonia Rating Scale (BFCRS). The x-axis represents time in weeks. The left y-axis represents average lorazepam dose in mg/day. The blue dashed line represents lorazepam dose and blue circles indicate the average value for the corresponding week. The orange solid line depicts BFCRS and the diamonds represent weekly average value of the BFCRS. Notably the * represents the endpoint for daily catatonia exams and catatonia was deemed resolved. Bars of various shade that indicate the duration and medication for clozapine (pink), memantine (burgundy), amantadine (purple). Zolpidem adjunct medication is represented by a dark blue bar. Formulations of mixed amphetamine salts are indicated with rectangular shades of green. Lastly, ECT sessions are shown at the bottom of the graph with gray bars aligning to where in the treatment bilateral-ECT occurred.

During the first week of admission, the patient’s Bush-Francis Catatonia Rating Scale (BFCRS) remained greater than 10, at which point clozapine was initiated to target psychotic symptoms. The final clozapine dose reached 37.5 mg before it was discontinued due to the development of fever, tachycardia, leukocytosis, elevated C-reactive protein (CRP), and concern for myocarditis. The patient was transferred to a local hospital for additional evaluation, where labs and electrocardiogram (ECG) were completed. Patient demonstrated elevated erythrocyte sedimentation rate, elevated CRP, normal white blood cell count, undetectable troponins, and no findings concerning for pericarditis or myocarditis that warranted further workup or hospitalization. Patient was transferred back to initial psychiatric hospital.

Shortly after initiation of clozapine, bilateral ECT was started three times per week to augment benzodiazepine treatment and facilitate resolution of catatonia. Although psychotic symptoms improved, catatonic features such as waxy flexibility and gegenhalten persisted despite 21 days of lorazepam at 24 mg TDD and 13 sessions of bilateral ECT. All 13 sessions lasted at least 30 seconds on electroencephalogram (EEG). Notably, there was a time in the middle of the clinical course when the BFCRS was 6, and the treatment team decreased lorazepam from 18 mg TDD to 10.5 mg in seven days. However, catatonia worsened at 10.5 mg, leading the treatment team to increase the TDD, which suggested continued dependence on high-dose benzodiazepine treatment.

In addition to high-dose lorazepam, memantine 5 mg twice daily was added for further augmentation in the treatment of catatonia, which consistently lowered the BFCRS for 5 weeks, reaching a score of 6 with waxy flexibility remaining present. Notably, memantine had to be discontinued after approximately 30 days because the patient developed a rash. Given the consistently low BFCRS, the treatment team began to lower lorazepam from 24 mg to 19.5 mg over the course of two weeks. However, catatonia worsened and the lorazepam increased back to 24 mg TDD. Overall, between days 49 and 122, the patient required lorazepam doses between 19.5 and 24 mg TDD. Amantadine was added on day 116 at 50 mg twice daily, however, BFCRS scores remained greater than 10. With respect to ECT, the patient started bilateral ECT on day 7 of admission and received his 38^th^ session on day 122. Remarkably, 37/38 ECT sessions had EEG seizures that were ≥ 25 seconds (with one of the 38 treatments being 18 seconds) without having a sustained effect on catatonia symptoms. It should be noted that while ECT did not completely lyse catatonia, the prolonged course was justified by worsening catatonia symptoms (particularly waxy flexibility, and posturing) on weekends when the patient did not receive ECT for two days, an evidence-based definitive treatment for catatonia with the aim of lowering the patients total daily dose of lorazepam. However, neither treatment was able to be tapered without worsening of catatonia within days.

Given the lack of response to gold-standard treatments and the temporal association with methamphetamine use, additional interventions were pursued. The patient was transferred to a medical-psychiatric unit at a general medical hospital within our hospital system to trial intravenous lorazepam, which was started at 16 mg TDD. However, patient found IV intolerable and removed it, therefore the patient was reinitiated on 24 mg PO after one day. The following day, mixed amphetamine salts extended-release 5 mg was initiated to target a hypothesized withdrawal-related hypodopaminergic state.

Within 48 hours of initiating mixed amphetamine salts, the patient’s BFCRS decreased from 15 to 6, and nine days later there was complete resolution of catatonia and the treatment team stopped tracking BFCRS. Zolpidem 5 mg was introduced as an additional treatment for catatonia. Over the following 49 days on the medical-psychiatric service, the patients regimen was adjusted to include lorazepam (tapered from 24 mg to 4 mg by discharge), zolpidem (titrated to 15 mg total; 5 mg in the morning and 10 mg at night), mixed amphetamine salts extended-release 10 mg, and mixed amphetamine salts immediate-release 5 mg. Furthermore, as the patient’s catatonia resolved there was concern for depression, and patient was started on mirtazpaine and titrated to 30 mg nightly. The patient was then transferred back to the initial psychiatric service he entered on admission for final medication adjustments and discharge planning.

The patient was discharged on this regimen with a plan to taper lorazepam by 0.25 mg per week in the outpatient setting. Notably, his catatonia improved without recurrence of psychotic symptoms, despite the absence of antipsychotic treatment and concurrent use of stimulant medications. Upon further evaluation during hospitalization, it became apparent that the patient had not experienced psychotic symptoms outside the context of substance use. Remarkably, all his prior hospitalizations for psychosis had been preceded by a period of methamphetamine use.

## Discussion

We present a case of a 32-year-old male with a questionable history of primary psychotic disorder, methamphetamine use disorder, and opioid use disorder, who developed severe, treatment-resistant catatonia in the context of methamphetamine withdrawal.

Evidence supporting methamphetamine withdrawal catatonia includes a comprehensive negative medical and neurologic workup, prolonged resistance to high-dose benzodiazepines and ECT, and a rapid and sustained clinical response following initiation of mixed amphetamine salts. The patient’s BFCRS remained in the double digits for four months despite treatment with high total daily doses of lorazepam (up to 24 mg daily), 38 sessions of ECT, and multiple augmentation strategies, before improving shortly after initiation of mixed amphetamine salts.

To our knowledge, methamphetamine withdrawal catatonia has not been previously described. While methamphetamine-induced catatonia has been reported, these cases typically respond to standard treatments such as benzodiazepines with or without ECT (6, 7, 14). Similarly, reports of clozapine withdrawal catatonia describe resistance to benzodiazepines until clozapine is reintroduced (8–10, 15), suggesting that disruption of dopaminergic and other neurotransmitter systems may play a critical role in catatonic pathophysiology.

The mechanism by which amphetamine salts may alleviate withdrawal-related catatonia remains unclear. Methamphetamine use is associated with increased synaptic dopamine levels, while chronic exposure has been linked to reduced D2 receptor density and dopaminergic dysfunction (16, 17). Aberrant D2 signaling has been implicated in features of catatonia such as rigidity and stupor (18). Additionally, catatonia has been associated with GABAergic hypofunction, glutamatergic dysregulation, and inflammatory processes (3, 19–21). Chronic methamphetamine use may disrupt these systems, creating a neurobiological environment conducive to catatonia.

One possible explanation is that amphetamine administration partially restores dopaminergic tone in a withdrawal-induced hypodopaminergic state, thereby facilitating resolution of catatonic symptoms. However, further research is needed to clarify the underlying mechanisms.

From a patient perspective, once catatonia improved, the patient expressed significant distress regarding the events leading to hospitalization. He described a longstanding pattern of methamphetamine use to induce manic and psychotic states, which he found subjectively desirable. He also reported concealing his substance use, which contributed to an initial diagnosis of primary psychotic disorder and subsequent treatment with antipsychotic medications, which he experienced as emotionally blunting. This, in turn, contributed to continued methamphetamine use.

Following recovery, the patient demonstrated a renewed commitment to sobriety. At discharge, he enrolled in a substance use treatment program and sought care with a dual-diagnosis psychiatrist to support ongoing recovery.

This case highlights a potentially novel presentation and treatment approach for catatonia. Methamphetamine withdrawal may represent an underrecognized etiology of catatonia, particularly given the prolonged psychiatric effects of stimulant use and the limitations of toxicology screening. Additionally, the use of stimulants in this context may appear paradoxical, given their association with psychosis. However, this case suggests that, in select circumstances, dopaminergic augmentation may play a therapeutic role in refractory catatonia.

Further study is warranted to better understand the relationship between stimulant use, withdrawal states, and catatonia, as well as the potential role of stimulant medications in treatment-resistant cases.

### Strengths and limitations

This case report has several limitations, the most obvious being the lack of objective or biochemical measure for the patient’s withdrawal state. It is difficult to prove that a patient several months removed from initial consumption of a substance remains in withdrawal. Additionally, the patient did show improvement to several augmentation strategies, but the medications had to be discontinued due to patient side effects. It remains unclear if clozapine or memantine would have resolved catatonia because the patient developed intolerable side effects as catatonia was improving. Strengths of the case report include how rapidly interventions were started and augmentations (medications and ECT) added. Furthermore, there was an extensive neurologic workup for encephalitis, meningitis, and lupus which included multiple brain MRI scans, several antibody markers, and consults to neurology. Lastly, the novel intervention of adding mixed amphetamine salts to the treatment course highlights the biggest strength of the case report. There was a reduction in BFCRS from 15 to 6 in 48 hours and led to a complete resolution of catatonia symptoms in 9 days. The mixed amphetamine salts was sustained as the lorazepam was decreased from 24 to 4 mg TDD, without recurrence of catatonia symptoms prior to discharge. Unlike the memantine reduction, which took 5 weeks, mixed amphetamine salts (mixed amphetamine salts ER and IR) showed a rapid decrease in BFCRS that was sustained through the lorazepam taper. This distinction in the speed and durability of response, while not definitive in a single case, is more consistent with a specific treatment effect than with coincidental recovery.

## Data Availability

The original contributions presented in the study are included in the article/supplementary material. Further inquiries can be directed to the corresponding author/s.

## References

[1] Tandon R Heckers S Bustillo J Barch DM Wolfgang G Gur RE . Catatonia in DSM-5. Schizophr Res. (2013) 150:26–30. doi: 10.1016/j.schres.2013.04.034 23806583

[2] Fink M Taylor MA . Catatonia: A Clinician’s Guide to Diagnosis and Treatment. New York, NY: Cambridge University Press (2006).

[3] Rogers JP Pollak TA Blackman G David AS . Catatonia and the immune system: a review. Lancet Psychiatry. (2019) 6(7):620–30. doi: 10.1016/s2215-0366(19)30190-7 31196793 PMC7185541

[4] Walther S Stegmayer K Wilson J Heckers S . Structure and neural mechanisms of catatonia. Lancet Psychiatry. (2019) 6(7):610–9. doi: 10.1016/s2215-0366(18)30474-7 31196794 PMC6790975

[5] Beach SR Gomez-Bernal F Huffman JC Fricchione GL . Alternative treatment strategies for catatonia: a systematic review. Gen Hosp Psychiatry. (2017) 48:1–19. doi: 10.1016/j.genhosppsych.2017.06.011 28917389

[6] Slavnic B Barnett BS McIntire S Becker R Sindhu S Vellanki KD . Methamphetamine-associated catatonia: Case series and systematic review of the literature from 1943-2020. Ann Clin Psychiatry. (2023) 35:167–77. doi: 10.12788/acp.0116 37459499

[7] Sullivan G Jiang S . Methamphetamine-induced catatonia. Prim Car Companion CNS Disord. (2022) 24:21c403211. doi: 10.4088/pcc.21cr03211 36126918

[8] Belteczki Z Ujvari J Dome P . Clozapine withdrawal-induced Malignant catatonia or neuroleptic Malignant syndrome: A case report and a brief review of the literature. Clin Neuropharmacol. (2021) 44:148–53. doi: 10.1097/wnf.0000000000000462 34132673

[9] Brailey J Bastiampillai T . Clozapine withdrawal Malignant catatonia in a medical intensive care unit setting. Asian J Psychiatr. (2020) 52:102043. doi: 10.1016/j.ajp.2020.102043 32311586

[10] Lander M Bastiampillai T Sareen J . Review of withdrawal catatonia: what does this reveal about clozapine? Transl Psychiatry. (2018) 8:139. doi: 10.1038/s41398-018-0192-9 30065280 PMC6068101

[11] Han B Compton WM Jones CM Einstein EB Volkow ND . Methamphetamine use, methamphetamine use disorder, and associated overdose deaths among US adults. JAMA Psychiatry. (2021) 78:1329–42. doi: 10.1001/jamapsychiatry.2021.2588 34550301 PMC8459304

[12] Kariisa M Scholl L Wilson N Seth P Hoots B . Drug overdose deaths involving cocaine and psychostimulants with abuse potential — United states, 2003–2017. MMWR Morb Mortal Wkly Rep. (2019) 68:388–95. doi: 10.15585/mmwr.mm6817a3 31048676 PMC6541315

[13] Ciccarone D . The rise of illicit fentanyls, stimulants and the fourth wave of the opioid overdose crisis. Curr Opin Psychiatry. (2021) 34:344–50. doi: 10.1097/yco.0000000000000717 33965972 PMC8154745

[14] Tomiyama G . Chronic schizophrenia-like states in methamphetamine psychosis. Japanese J Psychiatry Neurol. (1990) 44:531–9. doi: 10.1111/j.1440-1819.1990.tb01626.x 2074612

[15] Bilbily J McCollum B de Leon J . Catatonia secondary to sudden clozapine withdrawal: a case with three repeated episodes and a literature review. Case Rep Psychiatry. (2017) 2017:2402731. doi: 10.1155/2017/2402731 28396815 PMC5370482

[16] Ciccarone D . Stimulant abuse: pharmacology, cocaine, methamphetamine, treatment, attempts at pharmacotherapy. Prim Care. (2011) 38:41–58. doi: 10.1016/j.pop.2010.11.004 21356420 PMC3056348

[17] Wang GJ Smith L Volkow ND Telang F Logan J Tomasi D . Decreased dopamine activity predicts relapse in methamphetamine abusers. Mol Psychiatry. (2012) 17:918–25. doi: 10.1038/mp.2011.86 21747399 PMC3261322

[18] Seif P . Does dysregulation of the indirect pathway contribute to the pathophysiology of catatonia through neurotransmitter imbalance? Clin Neuropsychiatry. (2025) 22:229–42. doi: 10.36131/cnfioritieditore20250306 PMC12302819 40735384

[19] Ariza-Salamanca DF Corrales-Hernández MG Pachón-Londoño MJ Hernández-Duarte I . Molecular and cellular mechanisms leading to catatonia: an integrative approach from clinical and preclinical evidence. Front Mol Neurosci. (2022) 15:993671. doi: 10.3389/fnmol.2022.993671 36245923 PMC9558725

[20] Quinton MS Yamamoto BK . Causes and consequences of methamphetamine and MDMA toxicity. AAPS J. (2006) 8:E337–347. doi: 10.1007/bf02854904 16796384 PMC3231568

[21] Prakash MD Tangalakis K Antonipillai J Stojanovska L Nurgali K Apostolopoulos V . Methamphetamine: Effects on the brain, gut and immune system. Pharmacol Res. (2017) 120:60–7. doi: 10.1016/j.phrs.2017.03.009 28302577

